# The application of full-size three-dimensional individual printed model combined with three-dimensional digital demonstration can facilitate patient’s preoperative comprehension to robotic-assisted laparoscopic partial nephrectomy

**DOI:** 10.1186/s13741-022-00256-1

**Published:** 2022-06-28

**Authors:** Xiaobin Yuan, Xiaolei Liu, Qiang Jing, Fan Liu, Xuhui Zhang

**Affiliations:** 1 Department of Urology, First Hospital of Shanxi Medical University, Taiyuan, Shanxi China; 2 First College of Clinical Medicine, Shanxi Medical University, No. 56, Xinjiannan Road, Taiyuan, 030001 Shanxi China; 3 Shanxi Academy of Medical Sciences, Shanxi Bethune Hospital, Taiyuan, Shanxi China; 4 Third College of Clinical Medicine, Shanxi Medical University, Taiyuan, Shanxi China

**Keywords:** Reconstruction, Digital model, 3D printing, Renal tumor, Partial nephrectomy, Preoperative comprehension

## Abstract

**Background:**

In this study, it was aimed to evaluate the feasibility and effectiveness of full-size three-dimensional individual printed model (3D-IPM) based on computerized tomography (CT) reconstruction combined with 3D individual digital models (3D-IDMs) for improving the patient’s and their families’ comprehension levels of robotic-assisted laparoscopic partial nephrectomy (RALPN) preoperatively.

**Methods:**

Between January 2020 and January 2021, 37 patients underwent RALPN in our institution. 3D individual digital models (3D-IDMs) were reconstructed based on the data of computerized tomography (CT) scanning and full-size 3D-IPMs were fabricated correspondingly. For each patient and his/her closest accompanying immediate family member (CAIFM) (spouse or son/daughter), two semi-structured conversations were held by using CT films (1st conversation) and 3D-IPM combined with 3D-IDM demonstration (2nd one) respectively. The preoperative levels of comprehension were evaluated quantitatively by using a self-made preoperative comprehending score (PCS) in the patients and CAIFMs.

**Results:**

All the fabrications of full-size 3D-IPMs and all the operations were technically successful. The total PCS elevated significantly by presenting 3D-IPM combined with 3D-IDM demonstration compared with CT films (42.5 vs 35.5 in patients, *P* < 0.001; 42.9 vs 35.8 in CAIFMs, *P* < 0.001). Sub-PCSs in the evaluating aspects of renal anatomy, mass characteristics, the upcoming RALPN procedure, potential complication risks, and prognosis also showed a uniformed climbing pattern with the assistance of 3D-IPM+3D-IDM.

**Conclusion:**

The application of 3D-IPM presentation combined with 3D-IDM demonstration can improve the preoperative comprehension of patient and CAIFM to RALPN with more direct-viewing and verisimilar presentation, and can be used in RALPN patient education for increasing patients’ and their families’ cognitive empowerment.

**Supplementary Information:**

The online version contains supplementary material available at 10.1186/s13741-022-00256-1.

## Introduction

As the third most common malignancy in the genitourinary system, renal cell carcinoma (RCC) affects about nearly 3% of all adult malignancies (Ljungberg 2007) and led to 14,830 tumor-related death events and 73,750 new diagnosed cases in the USA last year (Siegel et al. 2020). For last three decades, the survival improvement of RCC mainly attributed to the timely implementation of surgical interventions (Hollingsworth et al. 2006), and after two decades of worldwide verification and clinical practice, LPN has now become the reference standard for surgical treatment of cT1 renal tumors (Smith 2016). Due to the asymptomatic and impalpable nature of cT1-staged renal mass, it is indispensable to use an auxiliary instructing tool to deliver the accurate and intensive information of oncology and surgical recommendation of LPN to the patients preoperatively. Conventionally, CT and magnetic resonance imaging (MRI) films have been playing important roles in the preoperative illustration to the patients. For most of the patients and their family without medical background, the indigestible cross-sectional images build up a formidable barrier to reach the clear and profound understanding of the oncological and therapeutic situation they are faced with. In spite of 3D-IDM reconstructed via computer-aided workstation, which can offer more detailed high-resolution information about the local vasculature, tumor mass, and its anatomical characteristics (Shao et al. 2013; Huang et al. 2012; Gu et al. 2006), represents a upgraded version of CT data application, these refined 3D images still cannot surpass beyond the limits of 2D screen, which inevitably result in the weakened presentation to the stereoscopic effects due to the inherent defect of plane displaying. Motivations for the maximal decision-making assistance and utmost treatment compliance from the patients and their families always give impetus to pursue an ideal tool featuring the highlights of vivid depicting and easily understood style. Currently, the technology of 3D printing indicates a rapid-updating and future-promising field in both of medical and biological innovations. We speculated that the face-to-face real physical model displaying (3D-IPM presentation) combined with the animated demonstration of planned surgical procedure (3D-IDM demonstration) can help to improve the patients’ preoperative education. From January 2020 to January 2021, we performed RALPN on 37 patients with cT1 renal tumors. We described our experience with the 3D-IPM fabrications to facilitate the preoperative communications in these cases.

## Methods

### General patient data

Between January 2020 and January 2021, 37 consecutive patients with cT1 renal tumors underwent RALPN in our institution. The ipsilateral glomerular filtration rate (GFR) was obtained with a camera-based method measuring the renal uptake of technetium Tc^99m^ diethylenetriaminepentaacetic acid, revealed that all patients had a normal-functional contralateral kidney. The detailed demographic data of the patients and CAIFMs are listed in Table 1.

**Table 1:**
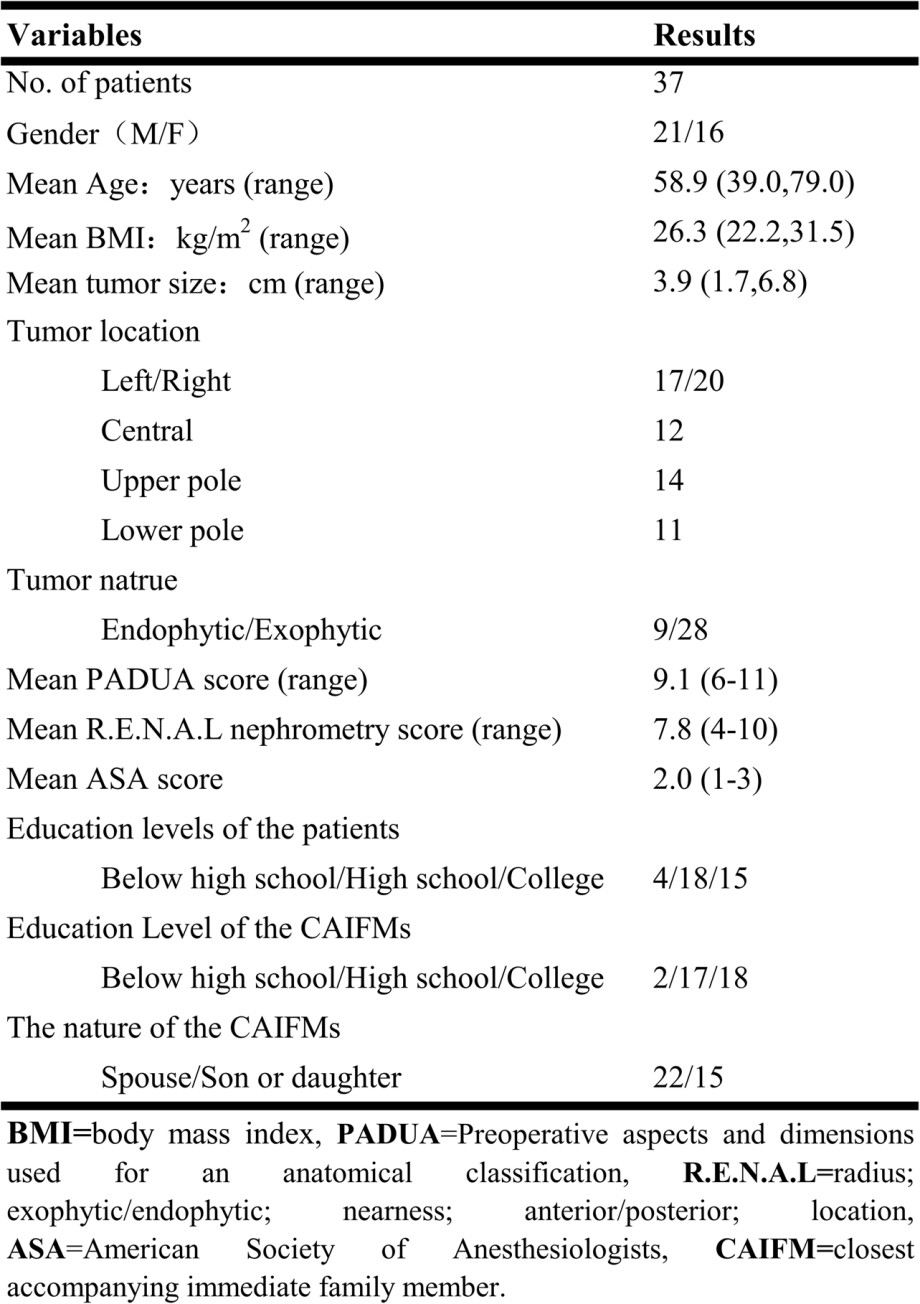
Demographic data of patients and CAIFMs

### CT scan and 3D-IDM reconstruction

The enhanced scanning including arterial phase, venous phase, and excretory phase after intravenous contrast administration was used to obtain the original information of tomo-sliced images on a dual-source 64-slice CT system (LightSpeed VCT, GE Healthcare, USA, 0.625 mm thickness) for all 37 enrolled cases.

The acquired CT data of three phases in the format of DICOM (Digital Imaging and Communications in Medicine) was processed to reconstruct the 3D-IDM of affected kidney by using both manual and automatic segmentation techniques on 3D medical image reconstructing and guiding system (3D-MIRGS), which was also described in a report of our relevant study previously (Wang et al. 2015).

**Fig. 1:**
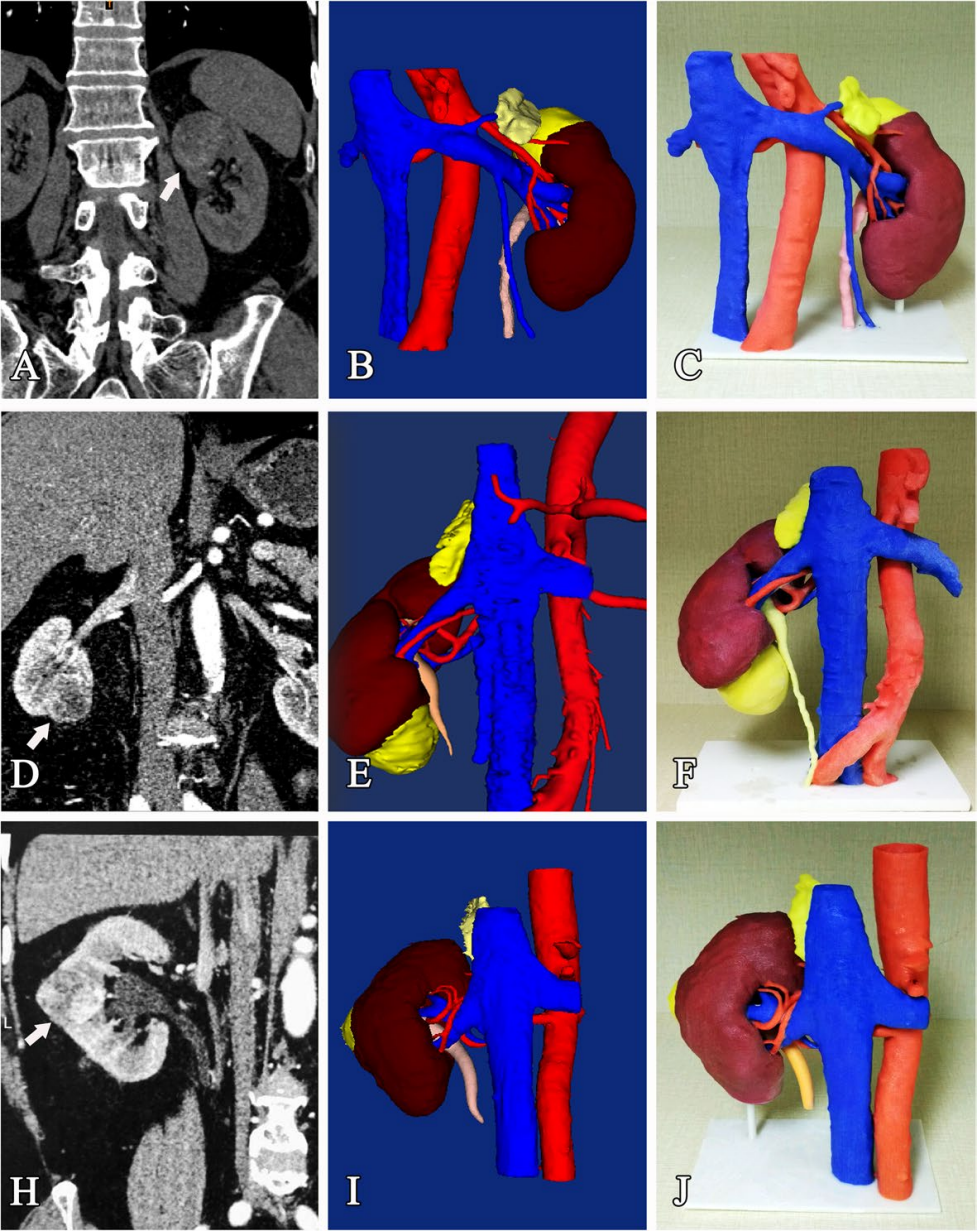
The CT-based 3D-IDMs and 3D-IPMs of the representative renal tumor cases (white arrow: the renal mass). **A**–**C** A 79-year-old male with a left-sided 4.8-cm renal mass on the upper pole (PUDUA score = 10P; R.E.N.A.L. nephrometry score = 8P). **D**–**F** A 53-year-old female with a right-sided 5.5-cm renal mass on the lower pole (PUDUA score = 11P; R.E.N.A.L. nephrometry score = 9P). **H**–**J** A 73-year-old male with a right-sided 4.0-cm renal mass in the mid part (PUDUA score = 10P; R.E.N.A.L. nephrometry score = 9P)

The anatomical region including the affected kidney, renal tumor mass, adrenal gland, and renal collecting system, as well as inferior vena cava (IVC), renal arteries, renal veins, and their branches were reconstructed simultaneously. Then, we applied rendering with different colors to distinguish the different vital structures (Fig. 1).

The reconstructed model can be observed from multiple directions and angles with the algorithm of object rotating for 360° along all axes. The system allows users to zoom in and out on the random angle with your free will for each part of the reconstructed anatomical structures. When it comes to the tumor that closed to the renal collecting system or ipsilateral adrenal gland, the users can click on the function icons to remove the tumor mass, turn the irrelevant renal parenchyma into light semitransparent style, and leave the tumor and renal collecting system with different heavy solid colors in the meanwhile, which can provide the intraparenchymal view to help the patients and CAIFMs to understand the spatial relationship between the tumor and concerned adjacent structures.

Maximal diameter of tumor mass, the location and length of targeted segmental renal artery, the optimal site of segmental renal artery clamping, and the volume of renal tissue were measured and calculated as the parameters of quantitative morphometry of the reconstructed 3D-IDM. Based on these parameters, an optimal surgical project was designed, which will be illustrated and demonstrated to the patents and CAIFMs preoperatively.

### The fabrication of 3D-IPM

According to the STL-formatted 3D-IDM provided as the printing template, a 3D printer (ProJet CJP 260C, 3D Systems Inc., USA) equipped with double print heads was used in the physical additive manufacturing. Core material named VisiJet PXL (3D Systems Inc., USA) was spread in thin layers over the build platform with a roller. After each layer of core material was spread, color binder was selectively jetted from inkjet print heads over the core layer, causing the core to solidify. The build platform lowered with each subsequent layer of core and binder until the high-resolution model was complete.

**Fig. 2:**
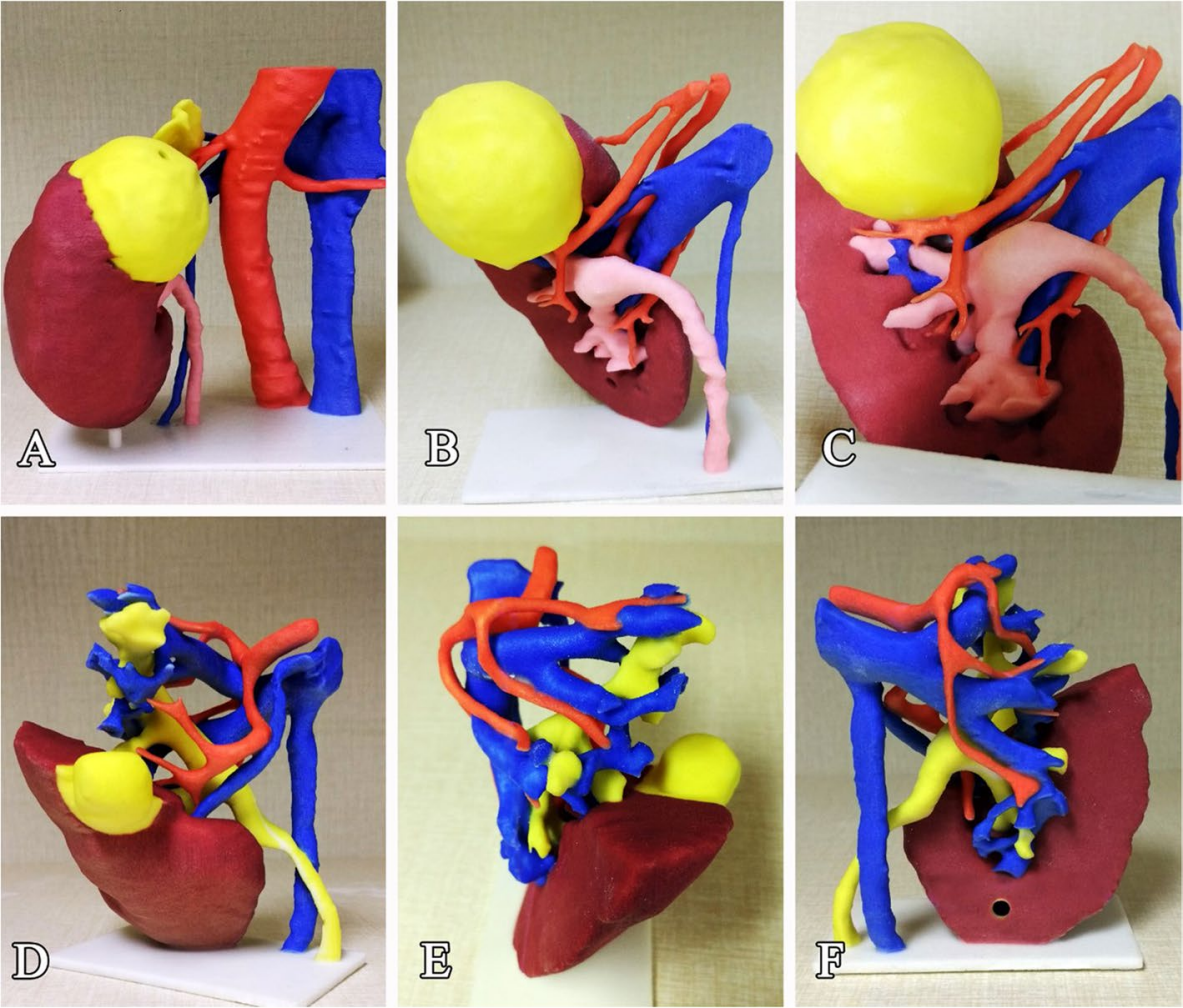
The distinct spatial relationship between the tumor and the nearby vital structures, including renal pelvic, ureter and renal vascular network, can be presented intuitively (**A**–**C**). The intrarenal sophisticated vasculature featured the targeted arterial branch can be presented in the life-sized 3D-IPM by using different colors (**D**–**F**)

The maximal printable size of this printer is 236 × 185 × 127 mm, which can satisfy the full-size fabrication demand of kidney perfectly (Fig. 2). A video demonstrating 3D-IDM demonstration and 3D-IPM fabrication accompanies this article (Additional file 1: Supplemental video).

### The preoperative conversations

For each patient and his/her CAIFM (spouse or son/daughter), two preoperative face-to-face semi-structured conversations with a single surgeon (*Xuhui Zhang*) were held by using patient’s CT films, the entity of 3D-IPM plus 3D-IDMs on IPAD screen as the auxiliary instructing tools separately. The patients’ and CAIFMs’ preoperative levels of comprehension were evaluated from five aspects as below: the renal anatomy, mass characteristics, the upcoming RALPN procedure, potential complication risks, and postoperative prognosis. A self-made 50-point questionnaire (Appendix 1) was used in the study.

Two days before the surgery, the first preoperative conversation was held. Three participants including the patient, his/her CAIFM and the designated surgeon were seated in a quiet and private consultation room to perform this conversation in the round-table style. The CT films were presented and the information of patient’s general health condition, the affected kidney, tumor and operation was delivered from the surgeon to the patient and CAIFM orally. After the communication, the patient and CAIFM were asked to complete the survey of the questionnaire independently. The day before the surgery, the second conference with the same pattern and the same members was held at the same place. The full-size entity of 3D-IPM was shown to the patient and CAIFM. With the free-will observation to the real physical model, the surgeon explained the kidney anatomy, tumor characteristics, the upcoming RALPN procedure, potential complication risks, and postoperative prognosis to the patient and CAIFM sufficiently. Meanwhile, in order to enhance the patient’s and CAIFM’s impression and understanding to the planned surgical manipulations, we demonstrated the vital steps of upcoming RALPN on 3D-IDM animatedly via an IPAD screen. The same questionnaire was completed by the patient and CAIFM separately after the conversation. All the survey sheets of 37 enrolled cases as well as their CAIFMs were collected and analyzed by a single investigator.

### Statistical methods

All data are reported as mean and range. The results were compared statistically by using Wilcoxon test, with *P* < 0.05 considered to indicate statistical significance. All the reported *P* values were two sided. The statistical analysis was accomplished with SPSS 18.0 (SPSS Inc., 2009, Chicago, IL, USA).

## Results

**Fig. 3:**
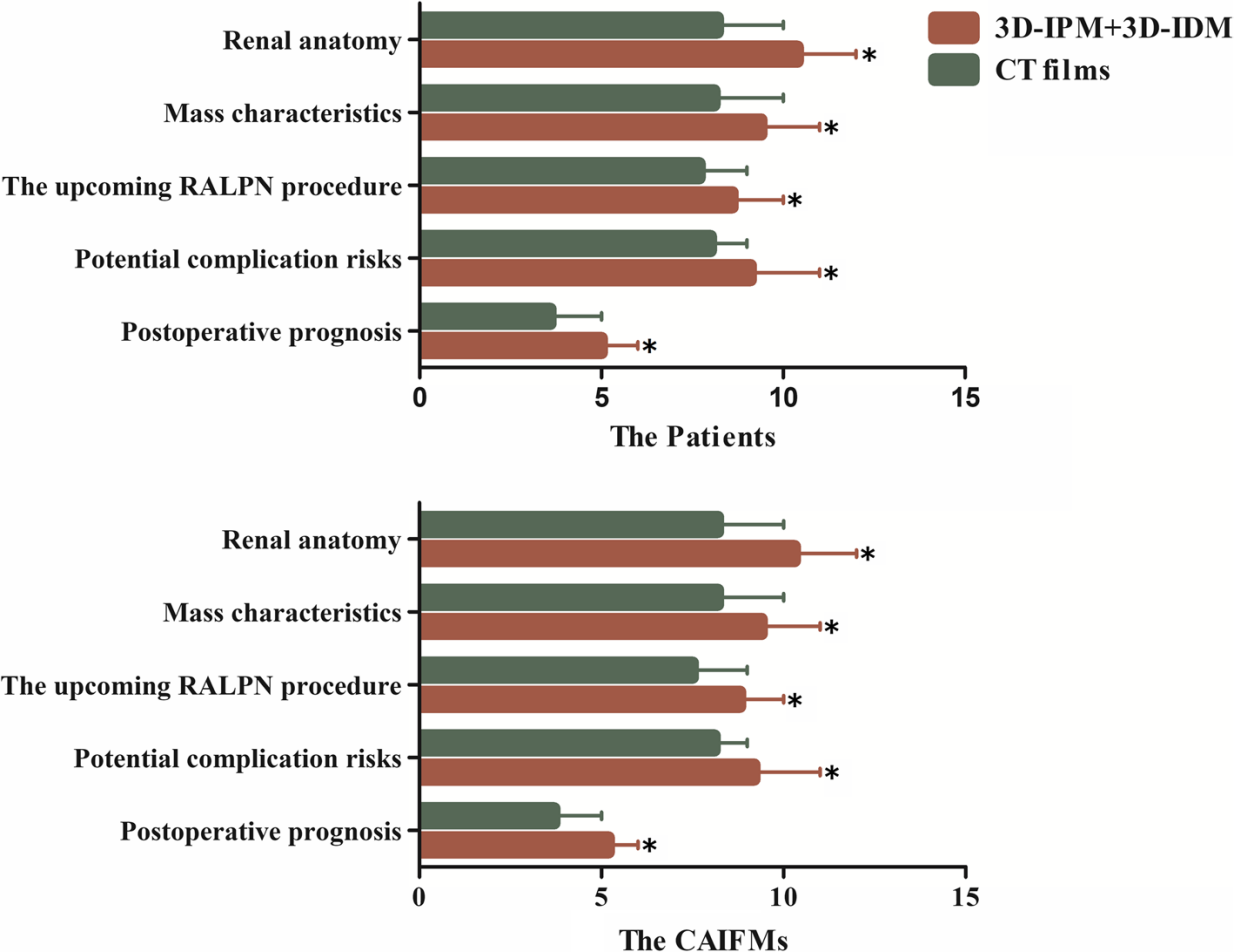
Patients’ and CAIFMs’ understanding assessment. Both in patients’ and CAIFMs groups, each Sub-PCS showed a climbing pattern with the assistance of 3D-IPM combined with 3D-IDM demonstration. (*P < 0.05, 3D-IPM + 3D-IDM compared with CT films)

All the fabrications of full-size 3D-IPMs were technically successful. Both in patient and CAIFM groups, the total PCS elevated significantly by presenting 3D-IPM combined with 3D-IDM demonstration compared with CT films (42.5 vs 35.5 in patient group, *P* < 0.001; 42.9 vs 35.8 in CAIFM group, *P* < 0.001). Sub-PCSs in the aspects of renal anatomy, mass characteristics, the upcoming RALPN procedure, potential complication risks, and prognosis also showed a uniformed climbing pattern with the assistance of 3D-IPM combined with 3D-IDM demonstration rather than CT films. The detailed data are listed in Table 2 and Fig. 3.

**Table 2:**
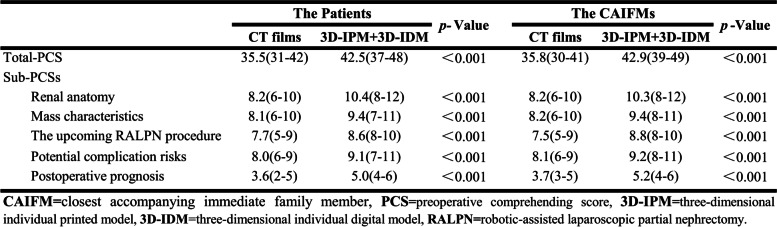
Patients’ and CAIFMs’ understanding assessment

## Discussion

In spite of the increasingly applications of 2D CT-sliced medical images, reconstructed digital models and 3D-printed entities in the preoperative interpretation of the oncological status and the followed surgical treatment the patient underwent, the direct objective benefit comparisons between the above three surgeon-patient communicating patterns in urological field were still rare.

In 2014, Silberstein at al. reported that they constructed 5 physical 3D models of renal units with suspected malignancies before surgery. The authors stated that patients and their families consistently considered that the models enhanced their comprehension of the renal tumor in relation to surrounding normal renal parenchyma and hilar structures and improved understanding of the goals of the surgery (Silberstein et al. 2014). However, the absence of quantitative evaluation for the comprehension improvement was the limitation of the study. In 2016, Komai Yoshinobu et al. (2016) and Zhang Yi et al (2016) also reported their outcomes of 3D-printing utilization in LPN respectively. Although this two studies all arranged brief rating scales to evaluate the understanding level of patients quantitatively, the nature of anecdotal reports without comparative arm, resulted in the deficiency of the study designing. The first quantitative and comparative study of 3D-printed renal model in the field of patient education was reported by Bernhard et al. (2016). They created life-size patient-specific 3D-printed models for seven LPN cases preoperatively and compared patients’ understanding level through a self-designed questionnaire before and after the presentation of 3D-printed models. The outcome indicated that the 3D printing can facilitate patient’s pre-surgical understanding of their kidney tumor and surgery. In spite of higher level of evidence this study provided, the ignorance of patient’s family member, who have significant referenced value as the important participant in the preoperative communication, was the imperfection of this study.

Inspired by the research of Bernhard et al, we designed this prospective pilot study. In our study, we reconstructed 37 patient-specific 3D digital models and fabricated their corresponding anatomically accurate, 3D-printed entities from CT data and evaluated the impact of this combination in the surgical comprehension of patients and their CAIFMs before the RALPN. According to our outcome data, the pattern of 3D-IPM presentation combined with 3D-IDM demonstration showed greater ratings in all 5 aspects including the renal anatomy, mass characteristics, the upcoming RALPN procedure, potential complication risks, and postoperative prognosis in both of patient and CAIFM groups, indicating that its higher cognitive level and better legible advantage for the patients’ and their CAIFMs’ oncological and surgical understandings compared with the traditional 2D CT film communication. 3D-IPM combined with 3D-IDM appeared to be the most appropriate mode of the patient and CAIFM understanding assistance. The direct-viewing experience delivered from the straightly observation and physically hand-touching of the targeted tumor mass and the adjacent vital structures on 3D-IPM, can obviously enhance the patients’ and their CAIFMs’ insight learning of the oncological anatomy.

Given that the static exhibition of 3D-IPM cannot provide sufficient information of the upcoming RALPN procedure, which can only rely on the verbal explanation traditionally, we introduced the supplementary demonstration of 3D-IDM. With the aid of this form of visual-animated illustration, patients and their CAIFMs can acquire a general idea and global profile of the upcoming RALPN procedure in advance more easily.

The exclusive assessment targeting at the group of patient’s family member is one distinctive feature of our study. In our study, all 37 enrolled patients agreed to inform their CAIFMs the oncological condition and the surgical plan. The irreplaceable physical-accompanying and emotional-supporting meanings to the patients, determine that the family member is an indispensable party of preoperative education. We believe that the higher level of compliance and coordination to the treatment can be achieved via the high-quality trilateral (surgeon-patient-CAIFM) preoperative conversations and subsequent informal bilateral (patient-CAIFM) discussions throughout the whole therapeutic cycle.

To the best of our knowledge, this is the first report of quantitative and comparative evaluation on the preoperative RALPN understanding for both of the patient and the CAIFM groups. The small number of enrolled cases and the character of single-surgeon series in single center are main limiting factors of our study. Another point need to be aware of is that the possible interfering and enhancing influence on the perception and cognition due to the repeated disease information delivering on the second conversation soon after the first one. We believe a multi-center, prospective, randomized, and controlled trial would emerge as the optimal design in the future. Besides, the issues of preoperative anxiety relieving and decision-making assistance did not addressed in our study. The questionnaire we designed did not cover this two important items. In our future serial research, the impacts on the preoperative anxiety and shared decision-making interventions will be compared quantitatively by using some reliable rating scales for anxiety assessment and treatment decision-making aids.

## Conclusions

The application of 3D-IPM combined with 3D-IDM demonstration can improve the preoperative comprehension of the patient and CAIFM to RALPN with more direct-viewing and verisimilar presentation. By increasing patients’ and CAIFMs’ cognitive empowerment, 3D-based technologies including 3D-IDM and 3D-IPM can be used as a favorable education method for patients undergoing RALPN and their CAIFMs in the future.

## Supplementary Information

**Additional file 1: Supplementary video. **3D-IDM demonstration and 3D-IPM fabrication accompanies this article.

**Additional file 2: Appendix 1.** Preoperative Comprehending Score Questionnaire.

**Additional file 3.** Research data.

## Data Availability

The datasets used and/or analyzed during the current study are available from the corresponding author on reasonable request.

## Abbreviations

3D-IPM: Three-dimensional individual printed model
CT: Computerized tomography
3D-IDMs: 3D individual digital models
RALPN: Robotic-assisted laparoscopic partial nephrectomy
CAIFM: Closest accompanying immediate family member
PCS: Preoperative comprehending score
RCC: Renal cell carcinoma
GFR: Glomerular filtration rate
3D-MIRGS: 3D medical image reconstructing and guiding system
IVC: Inferior vena cava
