# Supplementary figures and images for: The application of full-size three-dimensional individual printed model combined with three-dimensional digital demonstration can facilitate patient’s preoperative comprehension to robotic-assisted laparoscopic partial nephrectomy
Source: Perioper Med (Lond). 2022 Jun 28;11:22. doi: 10.1186/s13741-022-00256-1 (PMC9238097; doi:10.1186/s13741-022-00256-1)

**
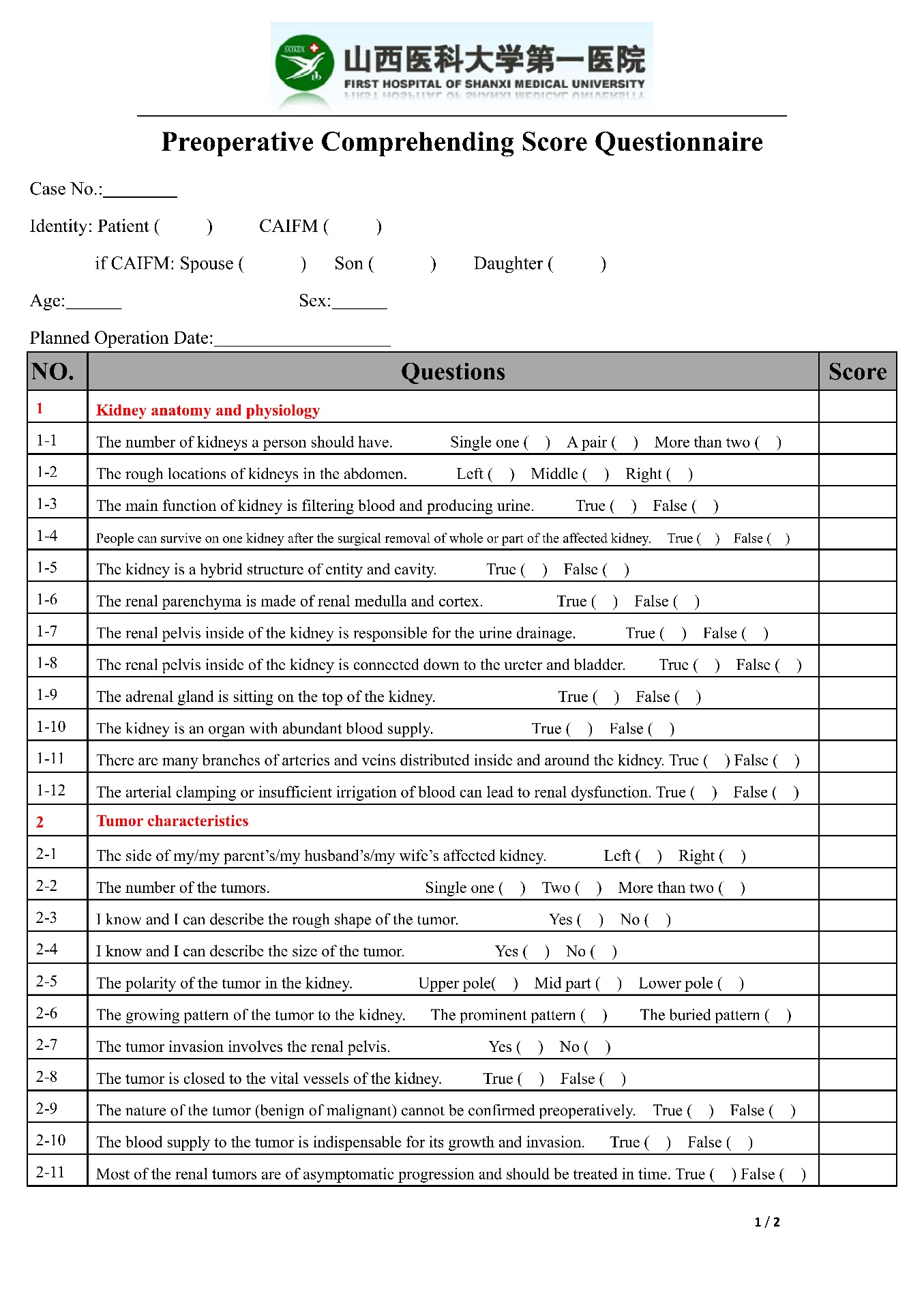
**

**
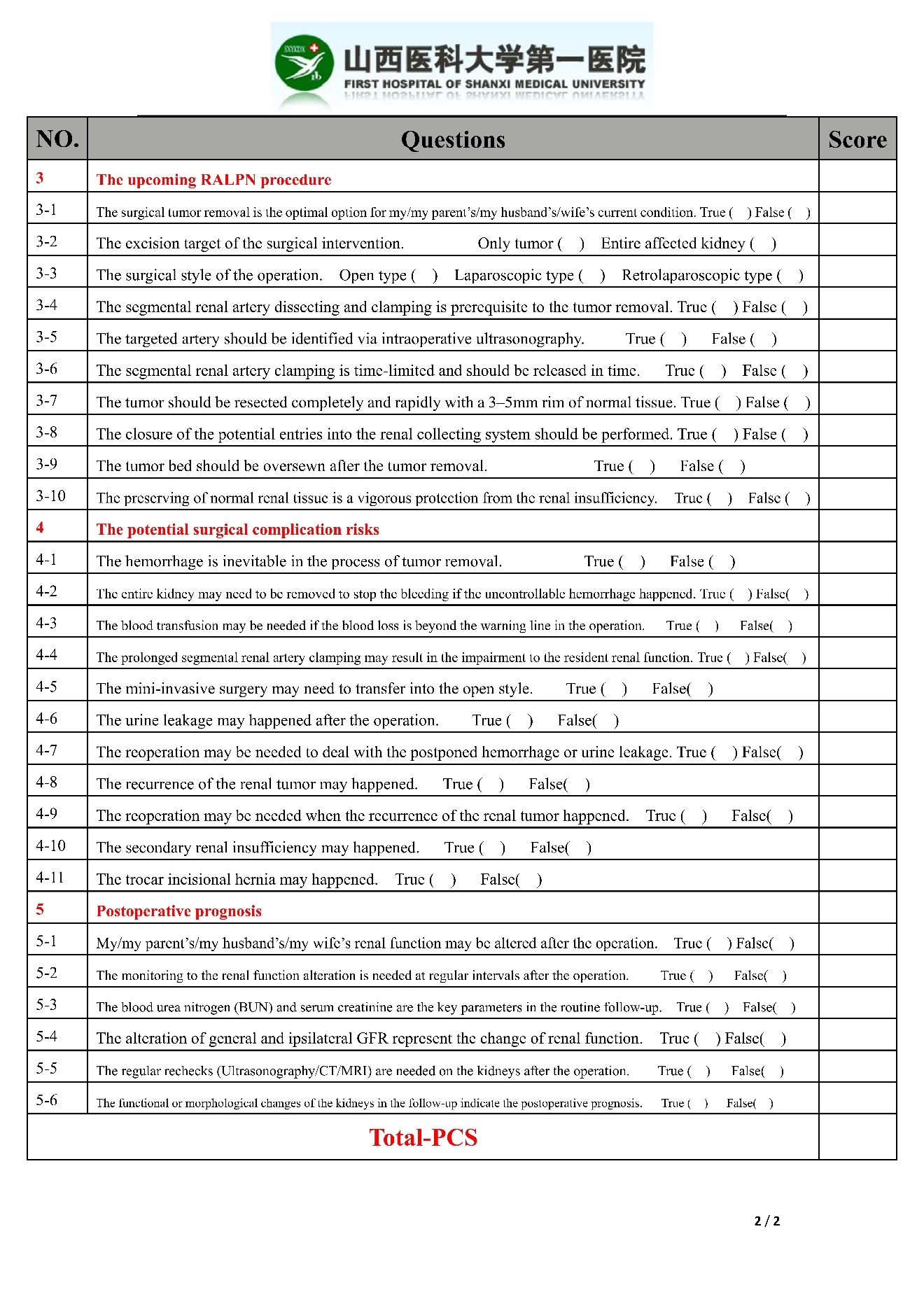
**

Supplement: Supplementary file 2 — Additional file 2: Appendix 1. Preoperative Comprehending Score Questionnaire. [file 13741_2022_256_MOESM2_ESM.docx]
